# Antidepressant in the Treatment of Chronic Pain: A Case Report of Adult-Onset Still's Disease

**DOI:** 10.7759/cureus.20180

**Published:** 2021-12-05

**Authors:** Ankit Jain, Krishna Priya Bodicherla, Rachana Vanaparthy

**Affiliations:** 1 Psychiatry & Behavioral Health, Penn State College of Medicine, Hershey, USA; 2 Psychiatry, Sri Devaraj Urs Medical College, Kolar, IND; 3 Research, Physicians for American Healthcare Access, Philadelphia, USA

**Keywords:** antidepressant, adult-onset still’s disease, chronic pain management, chronic pain, psychiatry & mental health

## Abstract

Adult-onset Still’s disease (AOSD) is a rare multisystemic autoinflammatory disease with symptoms, including spiking fever, evanescent rash, arthralgia or arthritis, sore throat, lymphadenopathy, hepatosplenomegaly, and myalgia. The prevalence of anxiety and depressive symptoms in rheumatological diseases is quite high, which impacts social as well as occupational functioning. Depression and anxiety are known to be the most common psychiatric comorbidities in patients with arthritis and other rheumatological disorders. Here, we report the case of an adult white female with AOSD who showed improvement in symptoms of AOSD with monoclonal antibodies and steroids; however, significant worsening of pain along with depression & anxiety were noted. With the use of antidepressant serotonin-norepinephrine reuptake inhibitor (SNRI), specifically duloxetine, our patient was able to experience improvement in depression, anxiety, and pain.

## Introduction

Chronic pain is a functional impairment that continues to be present after an injury and after healing has occurred. It causes discomfort, which often leads to the inability to perform various essential daily activities. Chronic pain treatment aims to help attain an individual's goal to achieve a manageable level of pain and have a good quality of life. Chronic pain is known to have significant psychiatric comorbidities. Comorbidity of depression and pain can affect individuals of any age, but it is more prevalent in the elderly affecting up to 13% of the elderly population. High rates of depression among patients with chronic pain and co-occurring other psychopathology can have important treatment implications [1]. Adult-onset Still’s disease (AOSD), first described in the early 1970s, is a rare multisystemic autoinflammatory disease with a prevalence of 1-10 per million people. Its symptoms include spiking fever, evanescent rash, arthralgia or arthritis, sore throat, lymphadenopathy, hepatosplenomegaly, and myalgia [2]. The prevalence of anxiety and depressive symptoms in rheumatological diseases is as high as 93%. Patients report symptoms of cognitive impairment in 66%, fatigue in 40%, and sleep disorders in 72% of the cases, which impact the social as well as occupational functioning [3]. Depression is one of the common psychiatric comorbidities in patients with arthritis and other rheumatological disorders. As per the diagnostic criteria, one-third of patients with rheumatologic conditions have comorbid major depression or dysthymia [4].

Here, we report the case of an adult white female with AOSD, who showed improvement in symptoms of AOSD with monoclonal antibodies and steroids; however, significant worsening of pain along depression and anxiety were noted. With the use of serotonin-norepinephrine reuptake inhibitor (SNRI), specifically duloxetine, our patient was able to experience improvement in depression, anxiety, and pain.

## Case presentation

A 36-year-old white female presented at our hospital for the management of worsening symptoms of depression and anxiety. Past medical history was significant for autoimmune thyroid disease and recently diagnosed AOSD. The patient had no previous psychiatric history but reported that she was experiencing increased feelings of sadness, worrying, hopelessness, fatigue, lack of concentration, increased muscle tension, feeling on edge, and poor sleep, most of which started when she was first diagnosed with AOSD.

Two years back, the patient was first diagnosed with systemic lupus erythematosus (SLE) based on a kidney biopsy read as consistent with class III lupus nephritis, for which she has been treated with mycophenolate mofetil 500 mg twice daily. Hydroxychloroquine was added, which resulted in the worsening of rash. The patient had used mycophenolate (for presumed lupus), tofacitinib, and adalimumab throughout her disease course over the years and did not find them helpful. The patient showed significant improvement of her symptoms after two courses of pulse dose steroids within about two months. Prophylactic therapy with antibiotics was continued due to the high dose steroid therapy. Her disease has remained largely under control on canakinumab 4 mg/kg subcutaneous every four weeks and prednisone 40 mg/day along with tofacitinib 5mg twice daily.

While some of her symptoms of AOSD like rash, and swollen lymph nodes, responded well to the above-mentioned treatment, she experienced significant worsening of anxiety and minimal change in her pain scores. The patient was started on hydroxyzine 50 mg every six hours as needed for anxiety and trazodone 100mg orally at bedtime for insomnia, but they were discontinued because of increased daytime sedation. She agreed to start duloxetine 30mg/day for anxiety, depression, and pain and was gradually titrated up to 90mg/day over three months. The importance of non-pharmacological methods was also discussed. These included progressive muscle relaxation, relaxation techniques for anxiety, and lifestyle counseling. On a scale of 0 to 10 on the numerical rating scale (NRS), the patient-rated her pain as eight, she scored 15 on the patient health questionnaire-9 (PHQ-9), and 13 on generalized anxiety disorder-7 (GAD-7). After three months of treatment, the patient’s symptoms of anxiety and depression showed significant improvement, as evidenced by a reduction in her PHQ-9 and GAD-7 scores to six and five, respectively. NRS score dropped to four.

## Discussion

With the emphasis on pathophysiology, physical symptoms, and pharmacologic interventions, comorbid depression and anxiety in rheumatological disorders are often missed. Healthcare providers should take into consideration that depressive disorders are common and frequently associated with chronic pain. The frequent association between pain and depressed mood can be explained by a common biological background that is involved in the modulation of both painful and emotional experiences [5]. The median disease duration is 10 years, i.e., half of the patients need treatment for more than 10 years. Somatic and psychosocial handicaps and prolonged pain exist, but complaints are not as permanent as in other rheumatic diseases [6]. It is also important to be mindful of the psychiatric side effects of immunomodulatory agents, which are often used to treat chronic pain conditions in rheumatological illnesses, as they can often have significant psychiatric side effects [7,8].

Patients with AOSD during disease flares usually suffer weeks of high spiking fever and joint pain, some have recurrent or polycyclic courses that cause repeated admissions, and a few patients with arthritis even developed permanent joint deformation, which leads to chronic pain and impaired ability. All of these increase the likelihood of anxiety and depression [9]. The pathogenic process of AOSD involves activating inflammatory cytokines, which in turn have been shown to contribute to the pathogenesis of anxiety and depression [10]. In recent years, duloxetine via 5-HT and NA has been found to modulate ascending spinal nociceptive neurotransmission via the descending inhibitory pain pathway, which is why duloxetine is used in chronic pain [11,12]. There have been some recent studies further establishing the role of anti-depressant medications in the treatment of chronic pain conditions in pediatric and adult populations [13]. There are many medications used to treat complex rheumatological diseases like AOSD, and clinicians should be aware of any drug-drug interactions between immunomodulators and other medications [14,15].

In the above-presented case, we found that while there was an improvement in symptoms of AOSD, for example like swelling of lymph nodes and rash with monoclonal antibodies and steroids. However, significant worsening of psychiatric symptoms like anxiety and depression along with pain were noted even with the continued use of monoclonal antibodies and steroids. With the use of SNRI antidepressants, specifically duloxetine, our patient was able to experience improvement in depression, anxiety, and pain. While this is one case of meaningful improvement in quality of life with the use of SNRI medication, more robust future studies need to be designed to explore the role of antidepressants in the management of AOSD.

## Conclusions

Over the last few years, immense progress has been made in the diagnosis and prognosis of AOSD. However, with the interrelation of pain and depression, it is important to have a close operation between rheumatologists and primary care to establish common therapeutic strategies and approaches to screen and address any concurrent psychiatric need. While the pathogenic process involves activating inflammatory cytokines, which in turn have been shown to contribute to the pathogenesis of anxiety and depression, more research is needed to figure out the mechanism of action of duloxetine and other antidepressants in addressing chronic pain in patients with AOSD. While it is plausible that antidepressant and anti-anxiety actions might have a big role in alleviating the psychological components of pain, there is increasing evidence of the effectiveness of antidepressant medications in alleviating chronic pain by themselves.
